# Evaluation of the SureX HPV genotyping test for the detection of high-risk HPV in cervical cancer screening

**DOI:** 10.1186/s12985-020-01417-8

**Published:** 2020-11-09

**Authors:** Baojun Wei, Ping Mei, Shengkai Huang, Xueting Yu, Tong Zhi, Guojing Wang, Xiaotian Xu, Lin Xiao, Xin Dong, Wei Cui

**Affiliations:** 1 Department of Clinical Laboratory, National Cancer Center/Cancer Hospital, Chinese Academy of Medical Sciences and Peking Union Medical College, No. 17 Panjiayuannanli, Beijing, 100021 People’s Republic of China; 2 Department of Pathology, Guangdong Provincial People’s Hospital, Guangdong Academy of Medical Sciences, Guangzhou, People’s Republic of China; 3 Department of Clinical Laboratory, Beijing Fengtai Youanmen Hospital, Beijing, People’s Republic of China

**Keywords:** High-risk human papillomavirus, HPV DNA test, Cervical cancer screening

## Abstract

**Background:**

The SureX HPV genotyping test (SureX HPV test), which targets the human papillomavirus (HPV) E6/E7 genes was compared with the Cobas 4800 and Venus HPV tests for detecting 14 high-risk HPV (HR-HPV) types in clinical referral and follow-up patients to evaluate its value for cervical cancer screening.

**Methods:**

Two different populations were enrolled in the study. The first population comprised 185 cases and was used for comparing the SureX HPV test (Health, China) with the Cobas 4800 test (Roche, USA). The second population comprised 290 cases and was used for comparing the SureX HPV test (Health, China) with the Venus HPV test (Zhijiang, China). Polymerase chain reaction (PCR) sequencing was performed for further confirmation of discordant results.

**Results:**

In the first population, the overall agreement rate was 95.6% for 14 high-risk HPV types. Eight discordant cases were confirmed by PCR sequencing, which showed that the agreement rates were 75.0% between the SureX HPV test and PCR sequencing and 25.0% between the Cobas 4800 test and PCR sequencing (*P* < 0.01). In the second population, the overall agreement rate was 95.5%. Thirteen discordant cases were confirmed by PCR sequencing, which showed that the agreement rates were 76.9% between the SureX HPV test and PCR sequencing and 23.1% between the Venus HPV test and PCR sequencing (*P* < 0.01). With cervical intraepithelial neoplasia grade 2+ (CIN2+) as the reference standard, the sensitivity values of the SureX HPV test and the Venus HPV test were 93.5% and 92.0%, (*P* > 0.05), while the specificity values were 43.3% and 46.7%, respectively (*P* > 0.05).

**Conclusion:**

The SureX HPV test had good consistency with both the Cobas 4800 and Venus HPV tests for 14 HR-HPV types. In addition, it avoided some false negatives and false positives. Therefore, the SureX HPV test can be used for cervical cancer screening.

## Background

Cervical cancer is one of the most common malignant tumors of the reproductive system in women. There were approximately 570,000 new cases of cervical cancer and 311,000 deaths worldwide in 2018 [1]. Persistent infection with high-risk human papillomavirus (HR-HPV) is recognized as the main cause of cervical cancer and precancerous lesions [2, 3]. HR-HPV DNA test for cervical specimens has been demonstrated as an effective approach to screen for cervical cancer and precancer, and has dramatically improved over the conventional cytology-based Pap smear [4]. Largely HR-HPV DNA test with polymerase chain reaction (PCR) is to detect the conserved region in L1 genes which code for the L1 capsid protein [5]. There is often an integration of HPV in the genome during progression from a low grade cervical lesion to cancer. And integration L1 expression can be lost. Therefore, this detection method for L1gene still needs improvement. But E6/E7 genes which encode oncogenic products remains always present, consequently, E6/E7 are pivotal in the development of cancer [6]. However, the degree of homology in this region limits digestion the detection of all HPV types [7]. Some studies have found that the detection of HPV E6/E7 DNA may be more accurate than the current DNA detection methods [8, 9]. The SureX HPV genotyping test (SureX HPV test) is a novel HPV DNA detection method using capillary electrophoresis fragment analysis technology to target the HPV E6/E7 genes. In the present study, the SureX HPV test was compared with the Cobas 4800 test [10] and the Venus HPV test for detecting 14 types of HR-HPV [11, 12] in clinical referral and follow-up patients to evaluate its value for cervical cancer screening.

## Materials and methods

### Study population

Two different populations were enrolled in this study. The first population comprised 185 cases with cervical lesions, which contained detectable HPV DNA and underwent cervical cytopathological evaluation in the Department of Pathology of Guangdong Provincial People’s Hospital between November 2017 and March 2018. The second population comprised 290 cases with cervical lesions, which contained detectable HPV DNA and underwent cervical histopathological evaluation in the Department of Clinical Laboratory of the National Cancer Center/Cancer Hospital between March 2018 and October 2018. The inclusion criteria included an age between 21 and 80 years, the absence of pregnancy, an intact cervix, no history of cervical lesions, and no history of chemotherapy, radiotherapy or surgical treatment.

### DNA extraction from cervical cells

In this study, for each patient, flushed the female genital tract with normal saline, probed a specimen collection brush into the cervix, turned the brush to take the cervical secretions after 5 s, and placed the brush in a 2 ml volume collection tube. The cervical specimens collection were performed by an experienced doctor as detailed for each test. And the cervical specimens were stored in a refrigerator (4 °C) and analyzed within 14 days.

For the SureX HPV test, total cellular DNA was extracted from cervical specimens using the extraction work station Smart LabAssist-16/32 (Taiwan Advanced Nanotech Inc., Taiwan) according to the manufacturer's instructions. For Cobas 4800 HPV test, HPV DNA was extracted from cervical specimens using the automatic nucleic acid extractor cobas × 480 DNA extractor (Roche Molecular Systems, Inc., USA). For the Venus HPV test, HPV DNA was extracted from cervical specimens using the automatic nucleic acid extractor Autrax workstation (Shanghai ZJ Bio-Tech Co., Shanghai, China). For the detection of 14 HR-HPV types, the three different method were used in accordance with the manufacturer's instructions. Negative and positive controls provided in the kits were included in each PCR test.

### SureX HPV genotyping test

The SureX HPV test (Health Gene Technologies, Ningbo, China) utilized amplification of target HPV DNA by multiplex polymerase chain reaction (PCR) and capillary electrophoresis to detect and genotype 25 HPV types according to the length of specific amplification fragments. The HPV types were identified by the test including HPV 6, 11, 16, 18, 26, 31, 33, 35, 39, 42, 43, 44, 45, 51, 52, 53, 56, 58, 59, 66, 68, 73, 81, 82 and 83. For the SureX HPV test, specifically designed primers were targeted on early genes E6, E7 and E1 of HPV types, plasmid pcDNA 3.1(+) (pcDNA for short) and human β-globin locused to make sure the length of the amplified PCR products were at least 3 nucleotides difference. So, via PCR amplification of target DNA, 27 targets could be identified by capillary electrophoresis in a single analysis according to the length of PCR products. The measure of β-globin served as a quality control mechanism to confirm that a negative result was not due to inappropriate sample collection or failure of DNA extraction. The internal control pcDNA could monitor the PCR process and ensured that the testing procedure had been properly performed. Therefore, a validated specimen should show the specific peaks of pcDNA and β-globin. The peak height of pcDNA ought to be equal to or greater than 500 RFU (≥ 500). When the peak of β-globin was absent indicating insufficient cervical cells, we suggested resampling should be conducted. For PCR amplification products (1 µl) subjected to capillary electrophoresis in an ABI 3500 Dx/3500xL Dx genetic analyzer, the cutoff value for determining specimens of HPV positive was:i) Signal of a HPV type ≥ 300 RFU; or ii) Peak area ratio (the ratio of the peak area of a HPV type to the peak area of pcDNA) ≥ 0.2.

### Cobas 4800 HPV test

The cobas4800 HPV test (Roche Molecular Systems, Inc., USA) used primers to define a sequence of approximately 200 nucleotides within the L1 region of the HPV genome, which specifically detected 14 high-risk types (16, 18, 31, 33, 35, 39,45, 51, 52, 56, 58, 59, 66, and 68). The cobas4800 test mainly included two processes. Firstly, HPV DNA was extracted through automated sample preparation, and then the HPV and β-globulin target DNA sequences were amplified by PCR primers. The amplified target DNA sequences were combined with their corresponding fluorescent probes. There were 4 types of fluorescent probes for detection, namely HPV16, HPV18, β-globulin and 12 other high-risk HPVs. The probes were labeled with different fluorescent dyes, and the subtype of HPV in the sample were determined by real-time monitoring of fluorescent signals. In addition, β-globin was used as an internal control (IC) to ensure a sufficient sample quantity for HPV DNA detection. If the cycle threshold (Ct) cutoff value for HPV16 was ≤ 40.5, a sample was considered HPV-positive; if the Ct cutoff value was > 40.5 and β-globin was effective, it was considered negative; otherwise, it was considered invalid. If the Ct cutoff value for HPV18 or any of the 12 other high-risk types was ≤ 40, a positive result was determined; and if the Ct cutoff value was > 40 and β-globin was effective, a negative result was determined; otherwise, the result was considered invalid.

### Venus HPV genotyping test

The Venus HPV genotyping test (Zhijiang Bio-Tech Co., Shanghai, China) was based on real-time fluorescence PCR technology and contained a specific ready-to-use system for the detection of 15 15 types of HR- HPV genotypes, including HPV 16, 18, 31,33,35,39,45,51,52,56,58,59,66, 68, 82. Detection of amplified HPV DNA fragments was performed in the fluorimetric channels FAM, HEX/VIC/JOE, TEXAS RED/Cal Red 610 and CY5 with the fluorescent quencher BHQ1. Human minibrain homolog (MNBH) was amplified as an internal control (IC) to indicate the presence of sufficient nucleic acid from the human MNBH gene. The Ct value was calculated. If the Ct value was ≤ 38.0, a sample was considered HPV-positive. If the Ct value of the IC was ≤ 32.0, and "undetermined" or "no CT" was displayed in the other channels, the sample was determined to be HPV-negative. If the Ct value was 38.0–40.0, the reaction was repeated. If the Ct value remained in this range and the amplification curve was a typical S-shape, the sample was considered HPV-positive; if the amplification curve was not a typical S-type, as the sample was considered HPV-negative.

### Sequencing

PCR sequencing was performed for further confirmation of discordant results. Sequencing reactions was targeted on type-specific E6/E7 gene and performed using the ABI PRISM BigDye Terminator V3.0 kit (Applied Biosystems) and analyzed in an ABI 3730 genetic analyzer (Applied Biosystems) at Sangon Biotech Co. (Shanghai). DNA sequences were then compared with the sequences of known HPV types using the Basic Local Alignment Search Tool from the National Center for Biotechnology Information website (https://www.ncbi.nlm.nih.gov/BLAST).

### Histological diagnosis

The cytopathological diagnosis was based on the nomenclature of the Bethesda system of cervical cytology. The histopathological diagnosis was classified according to the WHO histological criteria for cervical tumors and was used as the gold standard, with cervical intraepithelial neoplasia grade 2 (CIN2) and higher (CIN2+) considered positive. Cytological diagnosis was performed by the Department of Pathology, Guangdong Provincial People’s Hospital; pathological diagnosis, by the Cancer Hospital, Chinese Academy of Medical Sciences.

### Statistical analysis

All statistical analyses were conducted using SPSS 23.0. The consistency checks were evaluated by the Kappa (k) values. Using CIN2+ as a reference, the sensitivity, specificity, and area under the receiver operating characteristic (ROC) curve (AUC) were calculated. All differences with *P* values of < 0.05 (two-tailed) were considered statistically significant.

## Results

### Population 1

#### HR-HPV infection

The overall positive rates of the 185 cases for the SureX HPV test and the Cobas 4800 test were 72.4% (134/185) and 70.3% (130/185), respectively. Among the 185 cases, there were 12 NILM, 15 LSIL, 75 ASCUS, 43 HSIL+. In the different cervical cytopathological categories, no significant difference was observed in the positive rates of the 14 HR-HPV types between the SureX HPV test and the Cobas 4800 test (*P* > 0.05) (Table 1).

**Table 1:** Results of the SureX HPV and the Cobas 4800 tests by cytological category

	Total (%)	SureX HPV		Cobas 4800		*P* value
		Positive (%)	Negative (%)	Positive (%)	Negative (%)	
Total	185 (100%)	134 (72.4)	51 (27.6)	130 (70.3)	55 (29.7)	0.646
*Cytological category*						
NILM	12 (6.5)	4 (33.3)	8 (66.7)	4 (33.3)	8 (66.7)	1.00
LSIL	55 (29.7)	48 (87.3)	7 (12.7)	47 (85.5)	8 (14.5)	0.781
ASCUS	75 (40.5)	42 (56.0)	33 (44.0)	41 (54.7)	34 (45.3)	0.87
^a^HSIL+	43 (23.2)	40 (93.0)	3 (7.0)	38 (88.4)	5 (11.6)	0.458

*NILM* negative for intraepithelial lesion or malignancy, *LSIL* low-grade squamous intraepithelial lesion, *ASCUS* atypical squamous cells of undetermined significance, *HSIL* high-grade squamous intraepithelial lesion, *ACSH* high atypical squamous cells, *AGC* atypical glandular cell, *AIS* adenocarcinoma in situ, *SCC* squamous cell carcinoma

^a^HSIL+ includes HSIL, ACSH, AGC, AIS, and SCC

#### Agreement rate

The results of the SureX HPV test and the Cobas 4800 test were shown in Table 2. The validation showed good agreement between the two different methods for the 14 HR-HPV types. The overall agreement rate was 95.6% (177/185, Kappa = 0.894) (95% confidence interval [CI]: 0.812–0.961).

**Table 2:** HR-HPV distribution of SureX HPV and Cobas 4800 results

HPV genotype	SureX HPV	Cobas 4800	^a^SureX+/	SureX+/	SureX−/	SureX−/	Positive agreement (%)	Kappar	95% CI
			^a^Cobas+	Cobas−	Cobas+	Cobas−			
HPV16	33	38	33	0	5	147	86.84	0.913	0.832–0.982
HPV18	18	18	17	1	1	167	89.47	0.938	0.823–1.00
Other	100	98	95	5	3	85	92.23	0.915	0.851–0.968

^a^SureX, SureX HPV test; Cobas, Cobas 4800 test

Single HPV subtypes and multiple HPV subtypes were calculated together

There were 8 discordant results between the SureX HPV test and the Cobas 4800 tests; these results were confirmed by PCR sequencing, as shown in Table 3. Of the discordant results, 6 were positive by the SureX HPV test and PCR sequencing but negative by the Cobas 4800 test. The agreement rate was 75.0% (6/8) between SureX HPV test and PCR sequencing. Two cases were positive by the Cobas 4800 test and PCR sequencing but negative by the SureX HPV test. The agreement rate was 25.0% (2/8) between the Cobas 4800 test and PCR sequencing. The agreement rates were significantly different (*P* < 0.01).

**Table 3:** Discordance between the SureX HPV test and Cobas 4800 test

No.	SureX HPV	Cobas 4800	PCR sequencing	Cytology
1	16	Neg	16	LSIL
2	Neg	16	16	ASCUS
3	other^a^	Neg	other	ASCUS
4	other	Neg	other	ASCUS
5	other	Neg	other	ASC-H
6	other	Neg	other	HSIL
7	other	Neg	other	ASCUS
8	Neg	other	other	ASCUS

*Neg* negative

^a^12 HR-HPV is denoted “other” for Cobas 4800 test results

### Population 2

#### HR-HPV infection

Among the 290 cases, there were 33 diagnosed with normal cervix (11.4%), 56 CIN1(19.3%), 28 CIN2 (9.7%), 35 CIN3 (12.1%) and 138 cancer (47.6%), and the percentage of CIN2 was 30.7% and CIN 2+ was 69.3%.The overall positive rates of the 290 cases for the SureX HPV test and Venus HPV test were the same (78.3%, 227/290), as shown in Table 4. The positive rates of the 14 HR-HPV types did not differ significantly between the two methods in the different histopathological categories (*P* > 0.05).

**Table 4:** Results of the SureX HPV test and Venus HPV test by histopathological category

	Total (%)	SureX HPV		Venus HPV		*P* value
		Positive (%)	Negative (%)	Positive (%)	Negative (%)	
Total	290 (100%)	227 (78.3)	63 (21.7)	227 (78.3)	63 (21.7)	1.00
*Histological category*						
Normal	33 (11.4)	11 (33.3)	22 (66.7)	12 (36.4)	21 (63.6)	0.796
CIN1	56 (19.3)	28 (50.0)	28 (50.0)	30 (53.6)	26 (46.4)	0.705
CIN2	28 (9.7)	25 (89.3)	3 (10.7)	25 (89.3)	3 (10.7)	1.00
CIN3	35 (12.1)	34 (97.1)	1 (2.9)	30 (85.7)	5 (14.3)	0.088
CA^a^	138 (47.6)	130 (94.2)	8 (5.8)	130 (94.2)	8 (5.8)	1.00

^a^CA includes adenocarcinoma in situ (ACIS), squamous cell carcinoma (SCC), adenocarcinoma (AC) and adenosquamous carcinoma (ASC)

#### Agreement rate

The results of the SureX HPV test and the Venus HPV test were shown in Table 5. For both methods, HPV16 was the most frequently detected type, followed by HPV18 and HPV58 and HPV52. The concordance of the two methods for HPV 16, 18, 58, 52, 33, 51, 66, 35, 59, 56, and 39 was good, but was poor for HPV 45, 31, and 68. The overall agreement rate was 95.5% (277/290, Kappa = 0.838, 95% CI: 0.750–0.906, *P* < 0.01).

**Table 5:** HR-HPV distribution of SureX HPV and Venus HPV test results

HPV genotype	SureX HPV	Venus HPV	^a^SureX+/	SureX+/	SureX−/	SureX−/	Positive agreement (%)	Kappar	95% CI
			^a^Venus+	Venus−	Venus+	Venus−			
HPV 16	144	143	138	6	5	141	92.6	0.924	0.876–0.966
HPV18	28	32	27	1	5	257	81.8	0.889	0.786–0.970
HPV58	26	23	22	4	1	263	81.5	0.889	0.772–0.978
HPV52	18	20	17	1	3	269	81	0.887	0.758–0.976
HPV33	14	13	13	1	0	276	92.9	0.916	0.863–1.00
HPV51	10	9	9	1	0	280	90	0.946	0.775–1.00
HPV66	8	8	7	1	1	281	77.8	0.871	0.594–1.00
HPV35	4	3	3	1	0	286	75	0.855	0.329–1.00
HPV59	6	4	4	2	0	284	66.7	0.797	0.329–1.00
HPV56	7	8	6	1	2	281	66.7	0.795	0.491–1.00
HPV39	10	13	9	1	4	276	64.3	0.696	0.502–0.864
HPV45	5	3	2	3	1	284	33.3	0.493	− 0.862
HPV31	6	7	3	3	4	280	30	0.449	− 0.777
HPV68	4	3	0	4	3	283	0	− 0.012	− 0.025

^a^SureX: SureX HPV test; Venus:Venus HPV test

Single HPV subtypes and multiple HPV subtypes were calculated together

Thirteen results were discordant between the SureX HPV test and Venus HPV test; these results were confirmed by PCR sequencing, as shown in Table 6. Of the discordant results, 7 were positive by the SureX HPV test and PCR sequencing but negative by the Venus HPV test, and 3 were negative by the SureX HPV test and PCR sequencing but positive by the Venus HPV test. Thus, the agreement rate was 76.9% (10/13) between the SureX HPV test and PCR sequencing. Three cases were positive by the Venus HPV test and PCR sequencing but negative by the SureX HPV test. The agreement rate between the Venus HPV test and PCR sequencing was 23.1% (3/13). The agreement rates differed significantly (*P* < 0.01).

**Table 6:** Discordance between the SureX HPV test and Venus HPV test

No.	SureX HPV	Venus HPV	Sequencing	Histology
1	16	Neg	16	CIN3
2	16	Neg	16	CIN3
3	16	Neg	16	SCC
4	other^a^	Neg	other	SCC
5	other	Neg	other	SCC
6	other	Neg	other	CIN3
7	other	Neg	other	CIN2
8	Neg	16	16	AC
9	Neg	18	18	SCC
10	Neg	16/other	16/other	SCC
11	Neg	16	Neg	CIN1
12	Neg	other	Neg	SCC
13	Neg	other	Neg	CIN2

*Neg* negative

^a^12 HR-HPV, including HPV31, 33, 35, 39, 45, 51, 52, 56, 58, 59, 66, and 68, is denoted as “other”

### Sensitivity and specificity

**Fig. 1:**
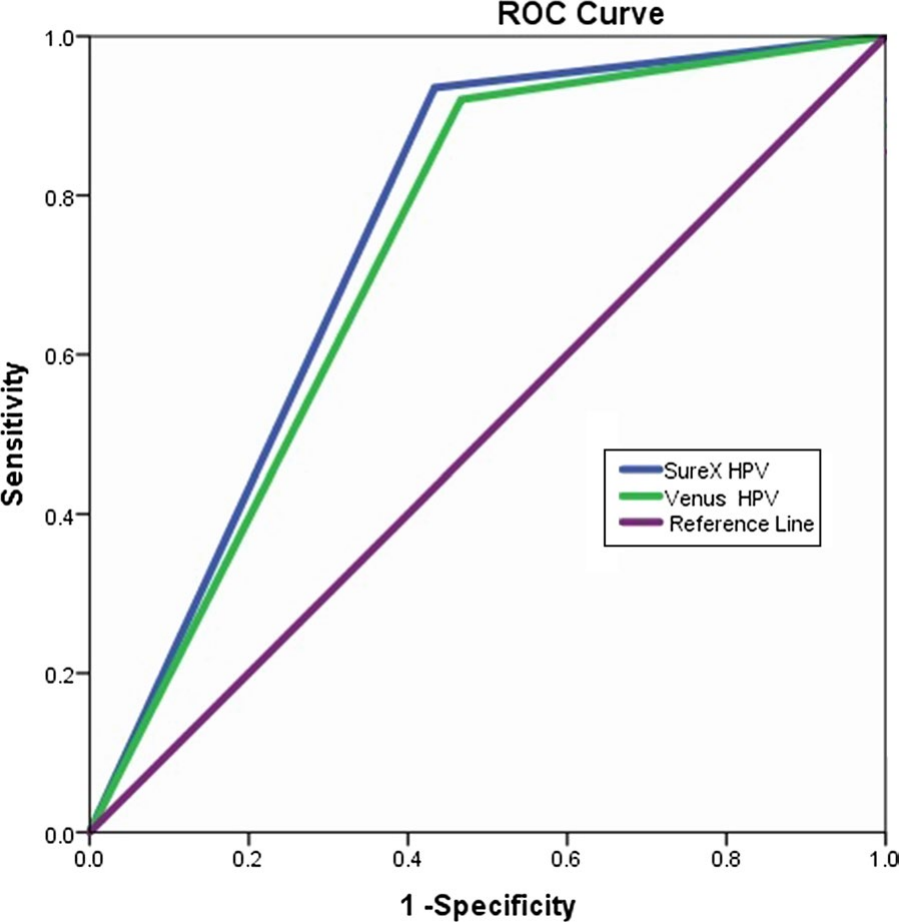
ROC curves for the SureX HPV test and Venus HPV test in CIN2+ lesions

With CIN2+ as the reference standard, receiver operating characteristic (ROC) curve analysis was used to calculate the sensitivity and specificity. The sensitivity values of the SureX HPV test and Venus HPV test were 93.5% and 92.0%, (*P* > 0.05), and the specificity values were 43.3% and 46.7%, respectively (*P* > 0.05). The AUCs for the SureX HPV test and Venus HPV test were 0.751 (95% CI: 0.683–0.819, *P* < 0.01) and 0.727 (95% CI: 0.658–0.96, *P* < 0.01), respectively (Fig. 1).

## Discussion

In the detection of HPV DNA PCR amplification, the selection of the target region and the design of the primers are particularly important for maximizing the amplification efficiency [13]. Because of the high conservation of HPV L1 DNA across genotypes, universal primers can be designed to amplify DNA from multiple genotypes; L1 DNA from different genotypes presents sufficient sequence differences to allow further analysis of specific genotypes by other methods [14]. Therefore, most HPV DNA tests currently on the market detect HPV L1 DNA [15]. The Cobas 4800 HR-HPV test uses HPV L1 DNA as an amplification target and can detect HPV16, HPV18, and 12 other HR-HPV types. The Venus HPV genotyping test also uses HPV L1 DNA as the detection target and can detect 15 HR-HPV subtypes. The Venus HPV test is widely used in China because of its specificity and sensitivity, which are better than those of similar products, and because it is easy to perform and inexpensive.

However, HPV L1 may be lost during the integration of HPV DNA into the host genome, and HPV tests based on L1 may lead to missed diagnoses of cervical cancer, which may affect the clinical sensitivity and positive predictive value of such tests [16, 17]. Research has shown that 0.3% of CIN2 and 3.94% of CIN3 lesions among HPV16-positive women were L1-negative [18]. Some researchers think that detection methods based on the E6/E7 gene are better than those based on the L1 gene [19, 20].

In this study, we compared the SureX HPV test with the Cobas 4800 and Venus HPV tests, which are widely used at home and abroad. The overall agreement rates were 95.3% (162/170, Kappa = 0.894) between the SureX HPV and the Cobas 4800 tests, and were 94.5% (274/290, Kappa = 0.838) between the SureX HPV and the Venus HPV tests. Therefore, good concordance was shown for detecting the 14 HR-HPV types between the SureX HPV and the Cobas 4800 tests and between the SureX HPV and the Venus HPV tests.

Persistent HR-HPV infection is the key factor for cervical cancer. The most common HPV genotypes causing cervical cancer are HPV 16 and 18 [21, 22]. The results of a large-scale multicenter epidemiological study in China showed that the most common types of HPV causing infection were HPV16 and HPV18, followed by HPV52 and HPV58 [23]. In this study, the results in the second population were consistent with those of that large-scale study.

To determine the actual HPV infection and show the accuracy of the SureX HPV test, PCR sequencing targeted on type-specific E6/E7 gene was performed to further confirm discordant results. In our study, 6 cases were positive by the SureX HPV test and PCR sequencing but negative by the Cobas 4800 test, and 7 were positive by the SureX HPV test and PCR sequencing but negative by the Venus HPV test. As both the Cobas 4800 and the Venus HPV tests target HPV L1 DNA, these 13 results may be false negatives due to missed detection of HPV L1 DNA. In addition, 2 cases were positive by the Cobas 4800 test and PCR sequencing, while 3 cases were negative by the SureX HPV test, and 3 cases were positive by the Venus HPV test and PCR sequencing but negative by the SureX HPV test. The reason for this discrepancy may be that the results of capillary electrophoresis in the SureX HPV test showed a peak height of pcDNA in the five samples of approximately 500 RFU, which may have resulted in low amplification efficiency because of low levels of HPV DNA in the cervical cell specimens. Although HPV DNA was extracted by three different automatic nucleic acid extractor in our study, the three automatic nucleic acid extractor were all magnetic bead methods and the three test had an internal control (IC) to indicate the presence of sufficient nucleic acid.

Additionally, studies had shown that although the HR-HPV DNA test had high sensitivity, the potential hazard of this method was that it could detect a large number of women with false-positive results, who were likely to have transient infections. After a few months, HPV is cleared naturally by the body without causing cervical cancer or precancerous lesions [24]. Three patients in our study were positive by the Venus HPV test but negative by the SureX HPV test and sequencing; these results may be false positives. The results of this study indicated that the agreement rate between the SureX HPV test and PCR sequencing were 75.0% (6/8) in the first population and 76.9% (10/13) in the second population; therefore, the SureX HPV test could be used for HR-HPV detection.

The method of screening cervical cancer and precancerous lesions by detecting HPV DNA was characterized by high sensitivity, while specificity was related to the positive rate of HPV. The specificity differed greatly among different populations [25]. With CIN2+ as the reference standard, the sensitivity values of the SureX HPV test (93.5%) was higher than the Venus HPV test (92.0%), and the specificity values of the SureX HPV test (43.3%) was lower than that of the Venus HPV test (46.7%). Some studies showed that the specificity of HPV DNA detection was lower than 50% [25, 26]. The AUC of the SureX HPV test were 0.751 (95% CI:0.683- 0.819, *P* < 0.01) and the Venus HPV test were 0.727 (95% CI: 0.658–0.96, *P* < 0.01). Therefore, there was reasonable consistency between the SureX HPV test and Venus HPV test.

In the study, there were 8 cases was negative to three different HR-HPV test and PCR sequence in HSIL+ or CIN2+ population. The possible reasons may be inappropriate sample collection, or the low copies of HPV DNA in the sample, or the patient factors, such as the patient had been treated but not informed.

Compared with previously developed and wisely used HPV detection methods such as real time PCR, the SureX HPV test described PCR-capillary electrophoresis method could achieve detection and identification of 25 HPV genotypes in one tube with targeting on specific oncogenic E6/E7. Normally it took around 6 h to have 96 specimens detected and reported if the capillary electrophoresis platform was 24-channel equipped. The testing cost is competitive with regular test methods.

## Conclusion

In summary, in this study, we compared a novel HPV genotyping test, the SureX HPV test, with the Cobas 4800 and the Venus HPV tests. The SureX HPV test had good consistency with the Cobas 4800 and the Venus HPV tests for detecting 14 HR-HPV types. In addition, the SureX HPV test could avoid some false-negative and false-positive results, and its sensitivity and specificity for pathological grade CIN2+ lesions was equivalent to that of the Venus HPV test. Therefore, the SureX HPV genotyping test is a novel method detecting HPV DNA, which utilizes PCR amplification and capillary electrophoresis to identify 25 HPV types in a single analysis, and it is an accurate, safe, and inexpensive HPV detection method. However, as the population selected for this study was the primary screening-positive population rather than the general population, further comparative analysis of these three methods through large-sample studies in the general population to provide a basis for the development of a large-scale cervical cancer screening strategy.

## Data Availability

Not applicable. All relevant data are within the paper.

## Abbreviations

AGC: Atypical glandular cell
AIS: Adenocarcinoma in situ
ASCUS: Atypical squamous cells of undetermined significance
ACSH: High atypical squamous cells
CA: Cancer
CI: Confidence interval
CIN: Cervical intraepithelial neoplasia
Ct: Cycle threshold
HR-HPV: High-risk human papillomavirus
HSIL: High-grade squamous intraepithelial lesion
IC: Internal control
LSIL: Low-grade squamous intraepithelial lesion
MNBH: Human minibrain homolog
NILM: Negative for intraepithelial lesion or malignancy
Neg: Negative
PCR: Polymerase chain reaction
ROC: Receiver operating characteristic curve (AUC)
RFU: Relative fluorescence units
SCC: Squamous cell carcinoma
SureX HPV test: SureX HPV genotyping test
